# Thalassemia does not significantly affect embryo ploidy outcomes in women undergoing IVF with preimplantation genetic testing

**DOI:** 10.3389/fcell.2025.1651060

**Published:** 2026-01-13

**Authors:** Li Fan, Wenjie Huang, Zhetao Li, Wugao Li, Liuyan Wei, Ni Tang, Liuying Nong, Jingjing Li, Huawei Wang

**Affiliations:** 1 Department of Reproductive Medicine, Guangzhou Women and Children’s Medical center Liuzhou Hospital, Liuzhou, Guangxi, China; 2 Department of Reproductive Medicine, Liuzhou maternity and Child Healthcare Hospital, Liuzhou, Guangxi, China; 3 Reproductive Genetics Department, The First Affiliated Hospital of Kunming Medical University, Kunming, Yunnan, China; 4 Guangxi Clinical Research Center for Obstetrics and Gynecology, Liuzhou, Guangxi, China; 5 Liuzhou Key Laboratory of Gynecologic Tumor, Liuzhou, Guangxi, China

**Keywords:** biopsied blastocysts, euploid, IVF, PGT-A, thalassemia

## Abstract

**Background:**

Thalassemia is a common autosomal recessive disorder that may impact reproductive health and embryo development. However, limited information is available regarding the impact of thalassemia carrier status on embryo euploidy, developmental competence, and embryo availability. Therefore, this study aims to evaluate the effects on women with thalassemia by analyzing data from women undergoing IVF with PGT-A.

**Methods:**

This retrospective cohort study included 2,110 women undergoing IVF with trophectoderm biopsy and PGT-A at Guangzhou Women and Children’s Medical Center Liuzhou Hospital between January 2019 and December 2024. Women were grouped by thalassemia status (955 with thalassemia vs. 1,155 without). Statistical analyses included the use of the Mann-Whitney U test for continuous variables, Chi-square test for categorical variables, and regression models (Poisson for the number of euploid embryos and linear for the proportion of euploid embryos). Adjustments were made for potential confounders, and statistical significance was set at a two-sided p-value <0.05.

**Results:**

After adjusting for key confounders including age, BMI, and AMH, no differences were observed between thalassemia and non-thalassemia women in embryo euploidy outcomes (adjusted RR for biopsied blastocysts: 1.06, 95% CI: 0.99–1.13; euploid embryos: 0.96, 95% CI: 0.86–1.08; proportion of euploid embryos: β = −0.03, 95% CI: −0.07–0.01). Although women with thalassemia initially showed higher numbers of biopsied blastocysts and euploid embryos in unadjusted analyses, these differences disappeared after adjustment. Further age-stratified analyses showed a slightly lower adjusted euploid proportion among thalassemia women aged 35–40 years (β = −0.07, 95% CI: −0.13 to −0.01), while no significant differences were observed in other age groups.

**Conclusion:**

Overall, no significant differences were observed after adjustment for age, BMI, and AMH; however, a mild decrease in euploid proportion was noted among thalassemia women aged 35–40 years. These findings suggest that thalassemia does not generally impair embryo chromosomal integrity but that careful counseling may be warranted in this specific age group.

## Introduction

Thalassemia, a common autosomal recessive genetic disorder prevalent in regions such as the Mediterranean, Southeast Asia, and the Middle East, includes α-thalassemia and β-thalassemia, both caused by defects in α-globin or β-globin chain synthesis, leading to anemia and iron overload (Langer et al., 1993; Tamary et al., 1993). Women affected by thalassemia often face reproductive health issues, such as chronic anemia and premature ovarian insufficiency (Tsilionis et al., 2024). Despite the significant impact of thalassemia on maternal health, most research has focused on transfusion management, obstetric complications, and fetal risks (Ruangvutilert et al., 2023; Li et al., 2025), with limited studies on how thalassemia carrier status might affect embryo health, specifically embryo euploidy and developmental competence during *in vitro* fertilization (IVF). This gap in knowledge highlights the need for further exploration, especially in the context of advancements in preimplantation genetic testing (PGT).

Over the past few decades, substantial advancements in PGT—from initially screening single-gene disorders (PGT-M) to detecting polygenic conditions and chromosomal abnormalities (PGT-A)—alongside improvements in embryo culture techniques such as blastocyst culture and vitrification, have significantly transformed modern *in vitro* fertilization (IVF). These technological advancements have enabled more precise embryo screening and selection, subsequently increasing clinical pregnancy rates and the probability of healthy live births. Consequently, the focus of patient counseling has shifted from merely achieving embryo transfer (ET) toward selecting embryos with the highest potential for healthy live births and minimal risk of complications (Ubaldi et al., 2019).

PGT-A is primarily indicated for patients with advanced maternal age or recurrent pregnancy loss (Rubio et al., 2013; Coonen et al., 2020). Numerous studies have documented that maternal age is a key determinant of embryo aneuploidy and reproductive outcomes (Franasiak et al., 2014). Recent research has further explored differences in embryo chromosomal abnormalities and embryo availability between IVF cycles employing combined PGT-M and PGT-A versus cycles using PGT-A alone (Stocker et al., 2022; Martel et al., 2024). However, while the combined use of PGT-A and PGT-M has increasingly been studied, existing literature primarily addresses embryo euploidy rates in general IVF populations, with limited systematic investigation into embryo euploidy outcomes among women with specific genetic disorders.

Given these technological advances, it remains unclear whether women with specific genetic disorders—such as thalassemia—experience differences in embryo chromosomal status or developmental potential during IVF. Women affected by thalassemia often suffer from chronic anemia and iron overload, potentially leading to premature ovarian insufficiency and endocrine dysfunction (Skordis et al., 2006; Ahmed et al., 2022). Despite these reproductive health implications, current thalassemia research mainly addresses transfusion management, obstetric complications, and fetal risks during pregnancy (Adler et al., 2021; Nassar et al., 2006; Charoenboon et al., 2016). Systematic data examining how thalassemia carrier status affects embryo euploidy, developmental competence, and embryo availability in IVF cycles utilizing PGT-A remain scarce.

Women with thalassemia face unique counseling challenges in assisted reproductive treatments, particularly regarding embryo health and genetic risks. Effective counseling significantly influences patient decision-making at all stages of treatment, ultimately impacting successful conception outcomes. Therefore, this study aimed first to evaluate differences in embryo euploidy rates between women with and without thalassemia undergoing IVF with PGT-A. Secondly, we assessed the likelihood of obtaining at least one transferable embryo between these two groups, stratified by maternal age, to better inform clinical practice and patient counseling.

## Materials and methods

### Study design and participants

This study was approved by the Ethics Committee of Guangzhou Women and Children’s Medical Center Liuzhou Hospital (No. 2024–238). Patients undergoing autologous IVF treatment at our center from 1 January 2019, to 31 December 2024, were included. Eligible cycles involved women with thalassemia confirmed by hemoglobin electrophoresis and genetic testing who underwent oocyte retrieval, trophectoderm biopsy, and PGT-A, with PGT-M additionally performed when both partners were carriers. Some diagnostic reports were obtained from external laboratories, and detailed subtype information (α- or β-thalassemia) was not consistently available. Women without thalassemia treated during the same period served as controls. Cycles involving donor oocytes or sperm, frozen-thawed oocytes, patients with anemia symptoms or other hereditary blood disorders, fresh embryo transfers, and non-thalassemia women whose partners were carriers of thalassemia were excluded.

Patient age was collected from official identification documents. Infertility diagnosis, ovarian stimulation protocol, and fertilization methods were recorded by the attending physician. Height and weight were measured on-site prior to oocyte retrieval, and BMI was calculated as weight (kg) divided by height squared (m^2^). Detailed embryo-related information, including embryo grading and the number of embryos biopsied, was documented by the embryology laboratory. Missing data were verified through electronic medical records. Serum hormone levels were retrieved from the clinical and genetic laboratory databases.

We analyzed data collected from IVF cycles that had already occurred. The ovarian stimulation protocols and embryo biopsy procedures were not specifically designed or modified for the purpose of this research. These treatments were part of the routine clinical care provided to patients, and the data was retrospectively analyzed to evaluate outcomes based on previously completed cycles.

## Ovarian stimulation protocols

All patients included in this study underwent standardized ovarian stimulation protocols established at our institution. Specific stimulation regimens, primarily involving GnRH antagonist or GnRH agonist protocols, were individualized by the attending physician based on ovarian reserve, patient age, and previous treatment responses. Follicular growth was monitored using transvaginal ultrasonography (7.5-MHz transvaginal probe, GE Voluson S10, United States), combined with serum measurements of estradiol (E2), luteinizing hormone (LH), and progesterone (P). Ovulation trigger was administered when at least two leading follicles reached a diameter of 17–18 mm (extended to 20–24 mm in letrozole or clomiphene cycles). Human chorionic gonadotropin (hCG), GnRH agonist (GnRH-a), or a combination of both was selected individually for triggering ovulation. Oocyte retrieval was conducted via transvaginal ultrasound approximately 35–37 h after the trigger. Intracytoplasmic sperm injection (ICSI) was primarily used to reduce the risk of DNA contamination when PGT was planned prior to fertilization, while conventional IVF was also performed in cases where PGT-A was added after fertilization based on patient preference. Fertilization was assessed on day 1 post-retrieval, with embryo morphology evaluated on days 3 and 5.

### PGT-A procedure

Embryos selected for biopsy were required to reach a minimum morphological grade of 3BC or higher based on the Gardner scoring system, as commonly applied in clinical practice and described in previous studies (Kirkegaard et al., 2012). Embryos with lower morphology or developmental arrest were not considered for biopsy. In the thalassemia group, the majority of patients underwent PGT-M to screen for pathogenic variants in α or β globin genes, and concurrently chose to perform PGT-A for comprehensive embryo assessment. In the control group, all patients underwent PGT-A alone based on standard clinical indications. Despite differences in indication, the biopsy procedure, embryo grading threshold, and reporting criteria for euploidy were consistent across both groups.

Blastocysts were biopsied at the trophectoderm stage on days 5–6 post-fertilization for PGT-A analysis. During biopsy, embryos were stabilized using a holding pipette. A hole of approximately 15–20 µm in diameter was made in the zona pellucida opposite the inner cell mass using an infrared laser (1.48 µm, ZILOS-tk system, Hamilton Thorne, Beverly, MA, United States). Embryos were further cultured for 4–6 h until 5–8 trophectoderm cells naturally herniated through the opening. Target cells were gently aspirated using a biopsy pipette and separated from the embryo using laser assistance under an inverted microscope with a micromanipulation system. Biopsied cells were washed in G-MOPS PLUS, transferred into 0.2 mL PCR tubes containing 2.5 µL lysis buffer, and stored, with the final wash solution serving as a negative control. Subsequently, next-generation sequencing (NGS) analysis was performed using Berry Genomics PGS kits (Beijing, China) on the Illumina MiSeq platform. Sequencing data were compared to the human reference genome, and chromosomal copy number variations were assessed using analysis software (e.g., BlueFuse Multi or platform-specific software), categorizing results into normal, aneuploid, or mosaic embryos.

### Statistical analysis

The primary outcomes were the number of euploid embryos per ovarian stimulation cycle and the euploid proportion (the proportion of euploid embryos among all biopsied embryos). Secondary outcomes included the number of usable embryos, defined as the euploid embryos determined to be suitable for transfer after PGT results. Baseline and cycle characteristics with non-normal distributions were summarized using median and interquartile ranges (IQR). No outcome variables were missing; other variables had missing data rates below 5%. Missing data were imputed using median values to mitigate the influence of outliers and ensure representative analyses.

Continuous variables were compared using the Mann-Whitney U test, and categorical variables were assessed by the Chi-square test. Poisson regression models were used to evaluate differences in the number of euploid embryos between the thalassemia and non-thalassemia groups, reported as relative risks (RR) with 95% confidence intervals (CIs). Linear regression models assessed differences in the proportion of euploid embryos, expressed as β coefficients with 95% CIs. All regression analyses included crude and adjusted models, with covariates including age, BMI, AMH, ICSI use, infertility diagnosis, stimulation protocol, total gonadotropin dose, oocytes retrieved, mature oocytes, and fertilized oocytes.

To evaluate potential effect modification, interaction terms were tested, including, thalassemia × age, and thalassemia × AMH. Post hoc subgroup analyses were performed to evaluate outcomes in each patient’s first PGT-A cycle. All statistical analyses were conducted using SPSS version 26.0 and R 4.3.2 (R Foundation for Statistical Computing, Vienna, Austria). Statistical significance was set at a two-sided p-value <0.05.

## Results

A total of 2,110 women were included in this study, comprising 1,155 women without thalassemia and 955 women with thalassemia. Significant differences were observed between the two groups regarding age, BMI, AMH levels, ovarian stimulation protocols, and number of retrieved oocytes (Table 1). Women without thalassemia had a significantly higher median age [39 years (interquartile range, IQR: 37–41)] compared to those with thalassemia [34 years (IQR: 30–38), p < 0.001]. BMI was significantly lower in the thalassemia group [21.64 (IQR: 19.99–23.60) vs. 22.48 (IQR: 20.81–24.84), p < 0.001]. Additionally, AMH levels were significantly higher among women with thalassemia [2.58 ng/mL (IQR: 1.59–4.28) vs. 1.99 ng/mL (IQR: 1.12–3.32), p < 0.001]. Genetic factors were the primary cause of infertility in women with thalassemia (72.7%), whereas diminished ovarian reserve (DOR) was predominant among women without thalassemia (46.3%, p < 0.001). Furthermore, women with thalassemia more frequently used GnRH agonist protocols (39.7% vs. 29.7%, p < 0.001) and had significantly higher numbers of retrieved oocytes, mature oocytes, and fertilized oocytes (all p < 0.001).

**TABLE 1:** Demographic and clinical characteristics among non-thalassemia and thalassemia women.

Characteristic	Non-thalassemia (n = 1155)	Thalassemia (n = 955)	p value
Age, y	39 (37–41)	34 (30–38)	<0.001
Age group, No. (%)			​
<35	193 (16.7)	563 (59)	​
35–37	133 (11.5)	137 (14.3)	​
38–40	414 (35.8)	147 (15.4)	​
41–42	226 (19.6)	56 (5.9)	​
>42	189 (16.4)	52 (5.4)	​
BMI	22.48 (20.81–24.84)	21.64 (19.99–23.60)	<0.001
AMH	1.99 (1.12–3.32)	2.58 (1.59–4.28)	<0.001
ICSI	1117 (96.7)	947 (99.2)	<0.001
Infertility diagnosis, No. (%)^b^			<0.001
Tubal factor	216 (18.7)	80 (8.4)	​
DOR or AMA	535 (46.3)	98 (10.3)	​
Genetic factor	0 (0)	694 (72.7)	​
Other	404 (35.0)	83 (8.7)	​
Stimulation protocol	​	​	<0.001
GnRH agonist	343 (29.7)	379 (39.7)	​
GnRH antagonist	348 (30.1)	230 (24.1)	​
High progesterone	429 (37.1)	302 (31.6)	​
Other	35 (3.0)	44 (4.6)	​
Total gonadotropin dose (IU)	2,475 (2,100–2,850)	2,400 (1950–2,850)	<0.001
Oocytes retrieved	10 (6–15)	13 (8–19)	<0.001
Mature oocytes	10 (6–15)	13 (8–19)	<0.001
Fertilized oocytes (2 P N)	8 (5–13)	11 (7–16)	<0.001

BMI, body mass index; AMH, anti-Müllerian hormone; ICSI, intracytoplasmic sperm injection; DOR, diminished ovarian reserve; AMA, advanced maternal age; IU, international units; 2  P N, two pronuclear. Data are presented as median (interquartile range) or number (%), as appropriate for non-normally distributed variables.

**FIGURE 1:**
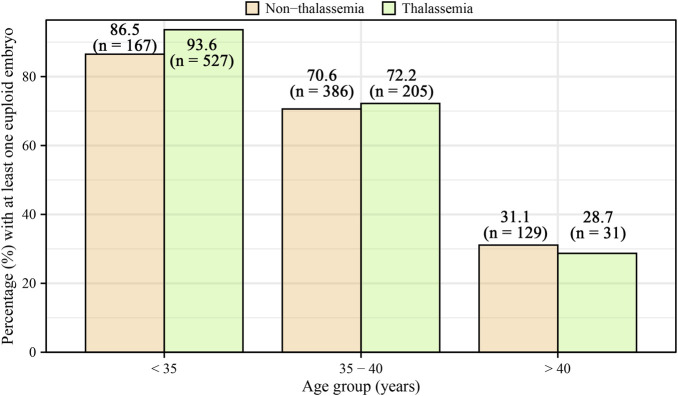
Age-stratified percentage of patients with at least one euploid embryo, categorized by age groups (<35, 35–40, >40) for both non-thalassemia and thalassemia women. The percentages for non-thalassemia were 86.5% (95% CI: 83.2%–89.9%) for <35, 70.6% (95% CI: 66.9%–74.3%) for 35–40, and 31.1% (95% CI: 26.4%–36.0%) for >40. For thalassemia, the percentages were 93.6% (95% CI: 91.3%–95.9%) for <35, 72.2% (95% CI: 65.4%–78.3%) for 35–40, and 28.7% (95% CI: 20.2%–37.2%) for >40.

Table 2 presents embryo biopsy and euploidy outcomes. Women with thalassemia had significantly more biopsied blastocysts [median 5 (IQR: 3–7)] compared to controls [median 3 (IQR: 2–5)], with a significantly higher unadjusted relative risk (RR = 1.32, 95% CI: 1.27–1.38). However, after adjustment for age, BMI, AMH, ICSI, infertility diagnosis, stimulation protocol, total gonadotropin dose, oocytes retrieved, mature oocytes, and fertilized oocytes, this difference became non-significant (adjusted RR = 1.06, 95% CI: 0.99–1.13). Similarly, the median number of euploid embryos was higher in women with thalassemia [2 (IQR: 1–4)] compared to controls [1 (IQR: 0–2)] in unadjusted analyses (RR = 1.91, 95% CI: 1.79–2.04), yet was not significant after adjustment (adjusted RR = 0.96, 95% CI: 0.86–1.08). The median proportion of euploid embryos was higher in women with thalassemia (0.50, IQR: 0.20–0.67) compared to controls (0.25, IQR: 0–0.50); however, this difference lost statistical significance after adjustment (adjusted β = −0.03, 95% CI: −0.07–0.01). The age-stratified proportion of patients with at least one euploid embryo is displayed in Figure 1. Supplementary Figures S1, S2 illustrate the distribution of cycles without any usable embryos and with all embryos usable, respectively.

**TABLE 2:** Association between women with thalassemia and embryo ploidy.

Ploidy	Non-thalassemia (n = 1155, 4,629 embryos)	Thalassemia (n = 955, 5,059 embryos)
Biopsied blastocysts	3 (2–5)	5 (3–7)
Unadjusted	1 (reference)	1.32 (1.27–1.38)
Adjusted	1 (reference)	1.06 (0.99–1.13)
Euploid embryos	1 (0–2)	2 (1–4)
Unadjusted	1 (reference)	1.91 (1.79–2.04)
Adjusted	1 (reference)	0.96 (0.86–1.08)
Euploid proportion^a^	0.25 (0–0.5)	0.5 (0.2–0.67)
Unadjusted	0.0 (reference)	0.14 (0.12–0.17)
Adjusted	0.0 (reference)	−0.03 (−0.07 to 0.01)

Data are presented as median (interquartile range). Poisson regression models, adjusted *a priori* for age, BMI, AMH, ICSI, infertility diagnosis, stimulation protocol, total gonadotropin dose, oocytes retrieved, mature oocytes, and fertilized oocytes were used to estimate the relative risk (95% confidence interval).

^a^ Linear regression models, adjusted *a priori* for the same variables, were used to estimate the ß coefficient (95% confidence interval).

Table 3 summarizes age-stratified embryo euploidy outcomes. Among women younger than 35 years, median counts were similar (both 5), but the distribution favored the thalassemia group (unadjusted RR = 1.08, 95% CI: 1.02–1.15), which was not significant after adjustment (adjusted RR = 1.08, 95% CI: 0.96–1.22). Similar patterns were observed for euploid embryos (unadjusted RR = 1.21, 95% CI: 1.11–1.32; adjusted RR = 0.96, 95% CI: 0.81–1.14). No significant difference was found in median euploid proportions (both 0.50, adjusted β = −0.02, 95% CI: −0.10 to 0.06). Women aged 35–40 years showed no significant differences in embryo biopsy or euploid embryo numbers, but a slightly lower euploid proportion was observed in the thalassemia group, becoming significant after adjustment (adjusted β = −0.07, 95% CI: −0.13 to −0.01). No significant differences emerged in women aged over 40 years Supplementary Table S1 indicated a significant negative interaction between thalassemia status and infertility categorized as “other” (β = −0.103, p = 0.033), suggesting the underlying infertility diagnosis may influence euploidy outcomes.

**TABLE 3:** Association between embryo ploidy and age subgroups in non-thalassemia and thalassemia women.

Age (y)/Ploidy	Non-thalassemia	Thalassemia
<35	n = 193	n = 563
Biopsied blastocysts	5 (3–7)	5 (4–8)
Unadjusted	1 (reference)	1.08 (1.02–1.15)
Adjusted	1 (reference)	1.08 (0.96–1.22)
Euploid embryos	2 (1–4)	3 (1–4)
Unadjusted	1 (reference)	1.21 (1.11–1.32)
Adjusted	1 (reference)	0.96 (0.81–1.14)
Euploid proportion	0.5 (0.25–0.67)	0.5 (0.33–0.70)
Unadjusted	0.0 (reference)	0.07 (0.03–0.11)
Adjusted	0.0 (reference)	−0.02 (−0.1 to 0.06)
35–40	n = 547	n = 284
Biopsied blastocysts	4 (2–5)	4 (3–6)
Unadjusted	1 (reference)	1.04 (0.96–1.12)
Adjusted	1 (reference)	0.97 (0.88–1.08)
Euploid embryos	1 (0–2)	1 (0–2)
Unadjusted	1 (reference)	1.02 (0.9–1.17)
Adjusted	1 (reference)	0.83 (0.69–1)
Euploid proportion	0.33 (0–0.5)	0.27 (0–0.5)
Unadjusted	0.0 (reference)	−0.03 (−0.07 to 0.02)
Adjusted	0.0 (reference)	−0.07 (−0.13 to −0.01)
>40	n = 415	n = 108
Biopsied blastocysts	3 (2–4)	3 (2–4)
Unadjusted	1 (reference)	1.07 (0.95–1.21)
Adjusted	1 (reference)	1.13 (0.98–1.3)
Euploid embryos	0 (0–1)	0 (0–1)
Unadjusted	1 (reference)	0.99 (0.71–1.35)
Adjusted	1 (reference)	1 (0.67–1.44)
Euploid proportion	0 (0–0.25)	0 (0–0.2)
Unadjusted	0.0 (reference)	−0.01 (−0.06 to 0.04)
Adjusted	0.0 (reference)	−0.02 (−0.08 to 0.04)

Data are presented as median (interquartile range). Poisson regression models, adjusted *a priori* for age, BMI, AMH, ICSI, infertility diagnosis, stimulation protocol, total gonadotropin dose, oocytes retrieved, mature oocytes, and fertilized oocytes, were used to estimate the relative risk (95% confidence interval). Linear regression models, adjusted *a priori* for the same variables, were used to estimate the ß coefficient (95% confidence interval).

A *post hoc* subgroup analysis limited to the first PGT-A cycles per patient confirmed the primary findings, revealing no significant differences in biopsied blastocysts (adjusted RR = 1.04, 95% CI: 0.97–1.12), euploid embryo numbers (adjusted RR = 0.92, 95% CI: 0.81–1.04), or euploid proportion (adjusted β = −0.04, 95% CI: −0.08 to 0). No significant interaction was found regarding the first PGT-A cycle (β = 0.072, p = 0.058).

## Discussion

This study is a large-scale, single-center registry-based cohort analysis aimed at comparing the euploidy rates between women with thalassemia and those without undergoing autologous IVF cycles combined with PGT-A. After adjustment for critical covariates such as age, BMI, and AMH, no significant differences in embryo euploidy outcomes were observed between thalassemia and control groups, suggesting that the initially higher unadjusted euploid counts in the thalassemia group were largely explained by differences in baseline characteristics. These adjusted results provide the most accurate interpretation of the data and form the basis of our clinical conclusions. Further subgroup analysis revealed a slightly lower euploidy proportion among thalassemia women aged 35–40 years, but no significant differences were observed in other age groups.

A systematic review involving 26 small-scale studies reported a reduction in transferable embryos from 57.5% to 37.2% following the addition of PGT-A to PGT-M, highlighting the potential increased risk of cycles without transferable embryos (Toft et al., 2020). Similarly, Stocker et. Demonstrated a decrease in embryo suitability for transfer from 69% to 41% when incorporating PGT-A into PGT-M treatment for autosomal recessive disorders (Stocker et al., 2022). Given that embryo availability relates not only to pathogenicity but also to euploidy, the reduced number of embryos available for transfer when utilizing more precise selection methods is unsurprising. It remains critical to consider whether the presence of single-gene disorders itself impacts embryo aneuploidy rates. Martel et al. further reported significantly lower rates of usable embryos among women under 37 years undergoing PGT-M compared to those receiving only PGT-A, though no significant differences in aneuploidy rates were found (Martel et al., 2024). These findings align with our observations, which indicated no significant association between thalassemia status and embryo euploidy rates. Nonetheless, the Martel et al. study had limitations, including the failure to explicitly exclude patients with single-gene disorders from the PGT-A-only group and the lack of stratified analysis by inheritance patterns or adequate adjustment for critical covariates such as age, BMI, and AMH (Martel et al., 2024). In contrast, our study focused explicitly on an autosomal genetic disorder—thalassemia—and excluded control participants and their partners who could potentially carry other genetic or hematologic conditions, thereby minimizing confounding factors. Moreover, we meticulously adjusted for multiple reproductive covariates such as age, BMI, and AMH levels, ensuring the robustness and reliability of our results. Subgroup analyses across various age ranges further demonstrated no substantial association between thalassemia and non-thalassemia women regarding embryo euploidy, particularly among groups younger than 35 years and older than 40 years.

Previous literature has suggested that oocyte quality significantly influences susceptibility to chromosomal segregation errors (Demyda-Peyrás et al., 2013). In women with thalassemia, iron overload leads to accumulation of non-transferrin-bound iron (NTBI) in ovarian tissues, which generates reactive oxygen species (ROS) via the Fenton reaction, inducing oxidative stress and damaging ovarian cells, potentially impairing oocyte developmental potential (Tsilionis et al., 2024). Furthermore, iron overload and oxidative stress disrupt hepatic and pancreatic functions, affecting hormone metabolism, exacerbating endocrine imbalances, and indirectly impairing the ovarian microenvironment, ultimately influencing oocyte quality (Singer et al., 2011; Castaldi and Cobellis, 2016). However, overall, women with thalassemia showed comparable chances of obtaining at least one euploid embryo compared to non-thalassemia women, with only minimal differences observed after adjustment. Small age-related variations were noted, with a slightly lower adjusted euploid proportion among women aged 35–40 years, whereas younger women showed similar or marginally higher euploid embryo counts. This mild decrease in the 35–40-year group may reflect the cumulative effects of chronic anemia–related oxidative stress, subtle age-associated declines in oocyte competence, and the increased metabolic demands of folliculogenesis in mid-reproductive age. Clinically, these findings suggest that women with thalassemia in this age range may benefit from more proactive counseling regarding expected embryo availability and the potential need for multiple stimulation cycles.

While this observation may partly reflect the younger age and better ovarian reserve parameters in the thalassemia group, the absence of significant differences in euploid outcomes after multivariable adjustment suggests that thalassemia itself does not impair, and may even preserve, chromosomal competence during embryo development. Mensia et al. similarly found no significant impairment in oocyte quality among women with thalassemia, despite lower ovarian reserves (Mensi et al., 2019). Our team’s previous research indicated that women with non-transfusion-dependent thalassemia exhibited superior oocyte maturity and fertilization outcomes compared to carriers, despite comparable baseline conditions (Fan et al., 2025). These observations suggest that thalassemia women might represent a systemic “low-risk phenotype,” analogous to observations in neurological and cardiovascular systems (Fallahzadeh et al., 2009; Dentali et al., 2011), though this hypothesis warrants further investigation.

A potential explanation for our findings could relate to differences in the reasons for seeking assisted reproductive treatments between thalassemia and non-thalassemia women, along with the generally younger age of the thalassemia group. These factors could influence embryo quality and euploidy outcomes. Previous studies also indicate that certain infertility conditions, such as recurrent pregnancy loss or previous IVF failures, typically exhibit lower euploidy rates compared to fertile populations (Kort et al., 2018). Therefore, thalassemia women may exhibit a tendency toward higher numbers of biopsied and euploid embryos. Nonetheless, after adjustment for covariates including age, BMI, and AMH, these differences were not statistically significant. Notably, the slightly higher proportion of women with thalassemia obtaining at least one euploid embryo may relate to higher fetal hemoglobin (Hb F) levels and lower LDL levels typically observed in these patients. Elevated Hb F levels effectively facilitate oxygen transport in hypoxic conditions (Ibrahim et al., 2009), and lower LDL concentrations have been positively associated with higher embryo quality and may indirectly enhance embryo implantation capacity (Wang et al., 2020; Herz et al., 1992).

This study’s strengths include being the first extensive investigation into PGT-A outcomes specifically in a thalassemia population, providing valuable empirical data on reproductive outcomes for these patients. All biopsy cycles were performed in the same laboratory, ensuring internal consistency and reliability. Our findings offer important clinical insights that aid physicians in counseling thalassemia patients and setting realistic expectations, fostering better physician-patient relationships. However, several limitations exist. First, retrospective data collection may introduce selection bias and data completeness issues, with incomplete control over all confounding factors. Although multivariable regression models, age-stratified analyses, and AMH interaction analyses were employed to adjust for key factors such as age and ovarian reserve, inherent differences in the clinical indications between the thalassemia and non-thalassemia groups—primarily genetic versus DOR/AMA—may still result in residual confounding. Notably, most thalassemia patients underwent IVF for genetic reasons rather than infertility; however, our study specifically aimed to assess whether thalassemia-related physiological factors affect embryonic chromosomal integrity during PGT-A, rather than reproductive potential itself. In addition, as many patients were referred from external centers after genetic screening, detailed genotype information (e.g., α-vs. β-thalassemia, or carrier vs. affected status) was not consistently documented. Second, as a single-center study, the generalizability of our findings might be limited, necessitating future multi-center studies for broader applicability validation. Lastly, clinical outcome data post-embryo transfer was not included, restricting the evaluation of actual improvements in clinical outcomes for thalassemia patients after euploid embryo transfer. Future studies should consider including clinical outcomes to further enhance understanding in this area.

## Conclusion

In conclusion, while women with thalassemia exhibited higher unadjusted euploid embryo counts, these differences were not significant after adjustment for age, BMI, and AMH. A mild but statistically significant reduction in euploid proportion was observed only among women aged 35–40 years. Overall, thalassemia does not appear to impair embryonic chromosomal integrity in IVF cycles with PGT-A, but cautious reproductive counseling may be advisable for women within this mid-age group.

## Data Availability

The original contributions presented in the study are included in the article/Supplementary Material, further inquiries can be directed to the corresponding authors.
