# Supplementary material for: Thalassemia does not significantly affect embryo ploidy outcomes in women undergoing IVF with preimplantation genetic testing
Source: Front Cell Dev Biol. 2026 Jan 13;13:1651060. doi: 10.3389/fcell.2025.1651060 (PMC12834768; doi:10.3389/fcell.2025.1651060)
Supplement: Supplementary file 1 [file Table1.docx]

**Supplemental Table 1.**  Interaction effects between thalassemia status and baseline covariates on euploid proportion.

| **Interaction** | **Estimate** | **Std Error** | **Z-value** | **p value** |
| --- | --- | --- | --- | --- |
| Age | 0.0044 | 0.0028 | 1.5640 | 0.1181 |
| BMI | 0.0034 | 0.0037 | 0.9110 | 0.3625 |
| AMH | -0.0045 | 0.0050 | -0.8920 | 0.3724 |
| ICSI | -0.0071 | 0.1061 | -0.0670 | 0.9467 |
| Infertility diagnosis, No. (%)^b^ |  |  |  |  |
| DOR or AMA | -0.0561 | 0.0465 | -1.2080 | 0.2274 |
| Genetic factor | 0.4671 | 0.5782 | 0.8080 | 0.4193 |
| other | -0.1030 | 0.0483 | -2.1300 | 0.0333 |
| Stimulation protocol |  |  |  |  |
| GnRH antagonist | 0.0037 | 0.0308 | 0.1200 | 0.9045 |
| High progesterone | -0.0028 | 0.0288 | -0.0980 | 0.9221 |
| Other | 0.0542 | 0.0645 | 0.8400 | 0.4011 |
| Total gonadotropin dose (IU) | 0.0011 | 0.0013 | 0.8000 | 0.4239 |
| Oocytes retrieved | -0.0019 | 0.0016 | -1.1990 | 0.2305 |
| Mature oocytes | -0.0019 | 0.0016 | -1.2130 | 0.2252 |
| Fertilized oocytes (2PN) | -0.0021 | 0.0019 | -1.1100 | 0.2671 |
