# Supplementary figures and images for: Thalassemia does not significantly affect embryo ploidy outcomes in women undergoing IVF with preimplantation genetic testing
Source: Front Cell Dev Biol. 2026 Jan 13;13:1651060. doi: 10.3389/fcell.2025.1651060 (PMC12834768; doi:10.3389/fcell.2025.1651060)

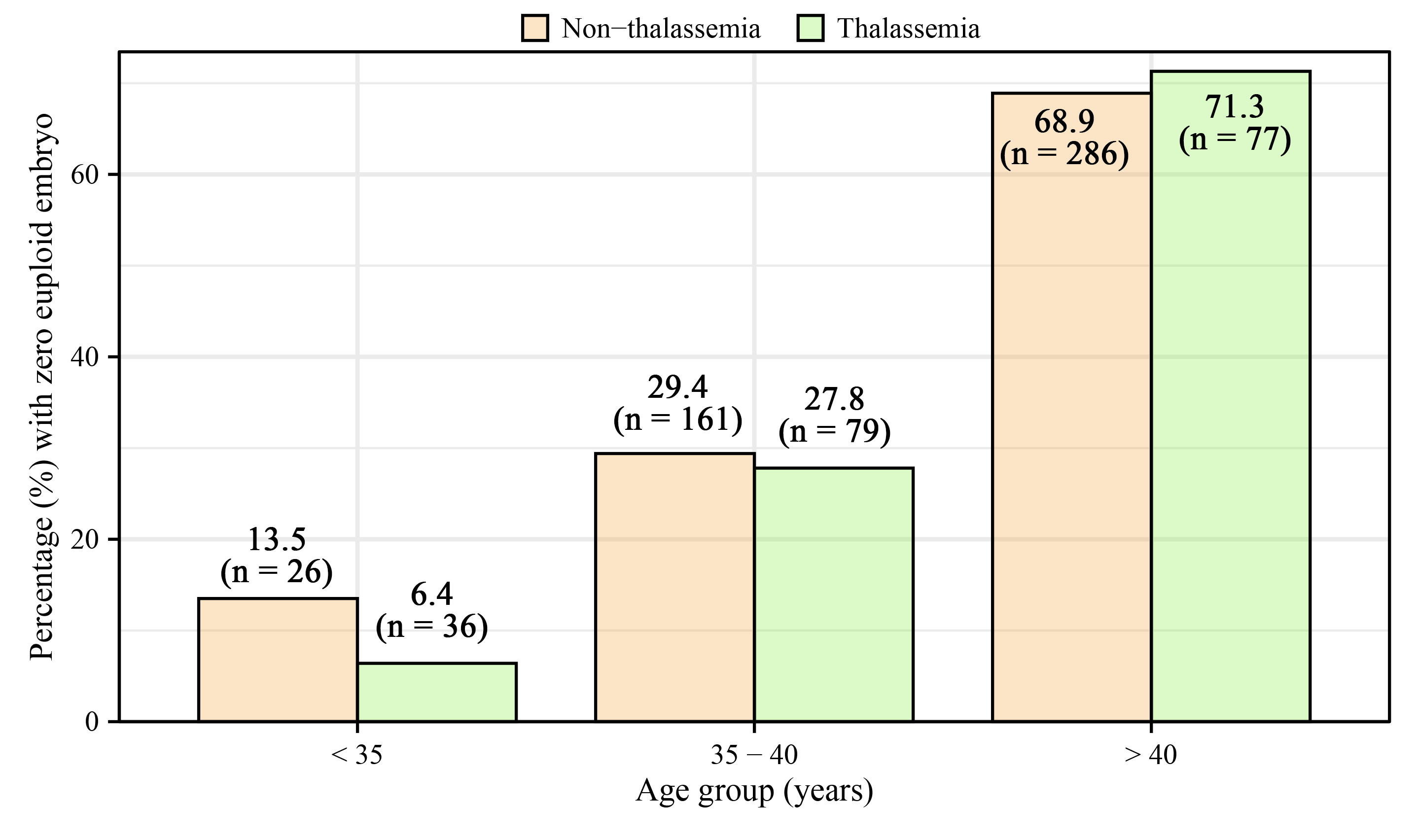

Supplement: Supplementary file 2 [file Image2.tif]

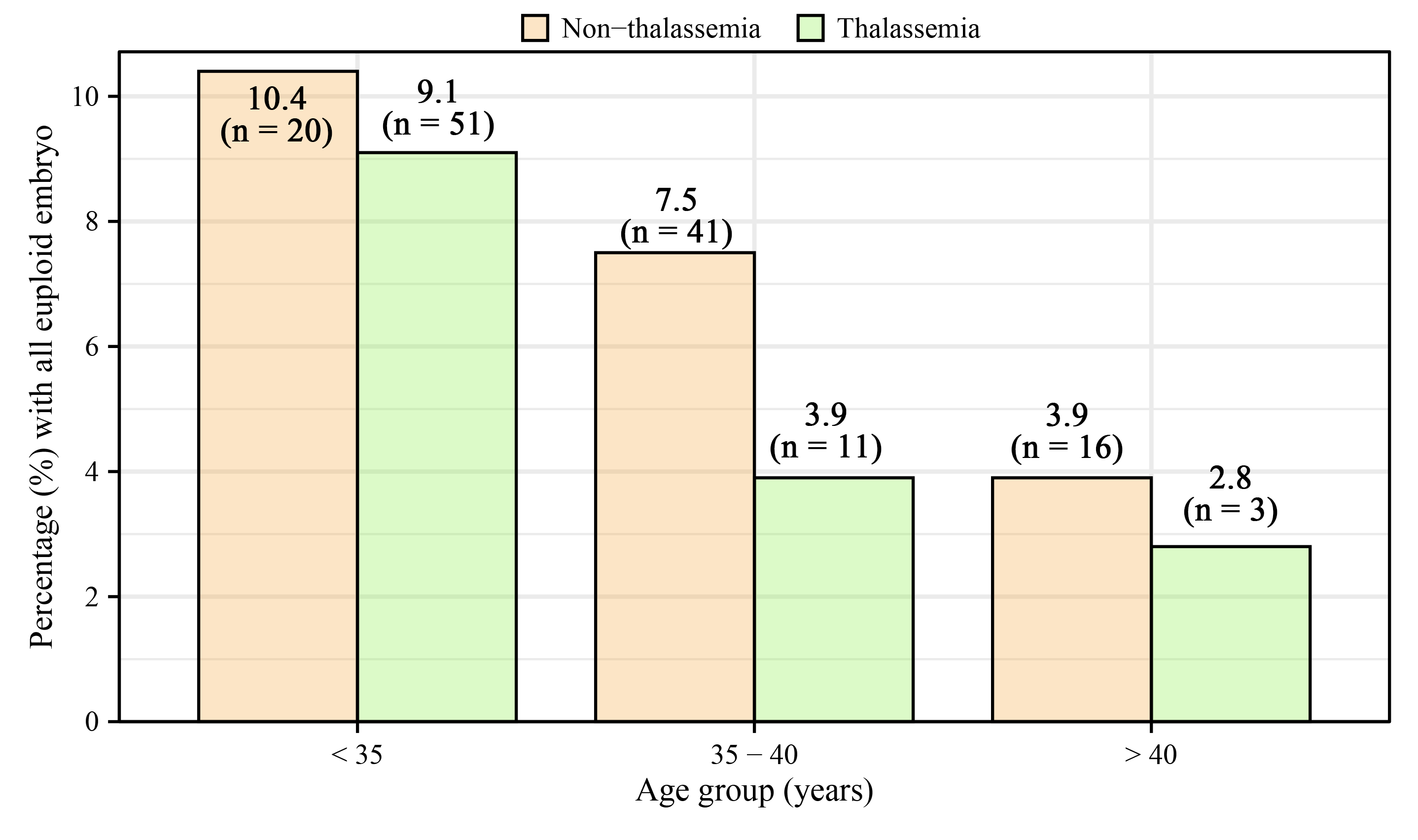

Supplement: Supplementary file 3 [file Image1.tif]
